# Neuroprotection Against a Panel of Toxicants via a Novel Analog of the Natural Product Fraxinellone

**DOI:** 10.1021/acs.chemrestox.5c00522

**Published:** 2026-04-22

**Authors:** Anna E. Bartman, Michael A. Garcia-Mares, Sarah E. Preston, Mersad Raeisi, Clarence D. Peiris, David B. C. Martin, Hans-Joachim Lehmler, Jonathan A. Doorn

**Affiliations:** † Department of Pharmaceutical Sciences and Experimental Therapeutics, College of Pharmacy, 4083 University of Iowa, 180 South Grand Avenue, Iowa City, Iowa 52242, United States; ‡ Department of Chemistry, College of Liberal Arts and Sciences, University of Iowa, 230 North Madison Street, Iowa City, Iowa 52242, United States; § Department of Occupational and Environmental Health, College of Public Health, University of Iowa, 145 North Riverside Drive, Iowa City, Iowa 52242, United States

## Abstract

Humans are exposed to a myriad of environmental pollutants, with recent evidence indicating several of these toxicants serve as risk factors for neurodevelopmental disorders and neurodegenerative diseases. Given this, there is a need for both interventional and protective strategies; however, of concern, the mechanistic targets of these environmental pollutants are variable or unknown in some cases. A prior report indicated that analogs of the natural product fraxinellone act as potent NRF2 activators, mitigating excessive reactive oxygen species (ROS) generation and Glu toxicity in vitro. Using one of the most effective fraxinellone analogs (i.e., analog 2) for NRF2 activation identified, we sought to determine the range of protection, in vitro, against a panel of neurotoxicants with varying mechanisms for adverse effects, including 6-hydroxydopamine (6-OHDA), organochlorine pollutants, and a fungicide. The data for analog 2 were compared to those for a structurally similar but inactive analog (i.e., analog 1). The dose–response for each toxicant with PC12 and SH-SY5Y cell lines was determined. Interestingly, the fraxinellone analog provided significant protection against all agents screened: 6-OHDA, dieldrin, benomyl, PCB52 hydroxy and sulfate metabolites, and rotenone. The extent to which the fraxinellone analog mitigated toxicity varied for each toxicant. In all cases, pretreatment with analog 2 significantly decreased total cellular ROS production, and in addition, generation of mitochondrial ROS via rotenone was mitigated. Furthermore, analog 2 provides some degree of restoration of cell viability following rotenone insult. In summary, our data indicate that an analog of the natural product fraxinellone potently inhibited ROS production and toxicity, thereby protecting cells against a panel of agents with varying mechanisms from adverse outcomes.

## Graphical abstract

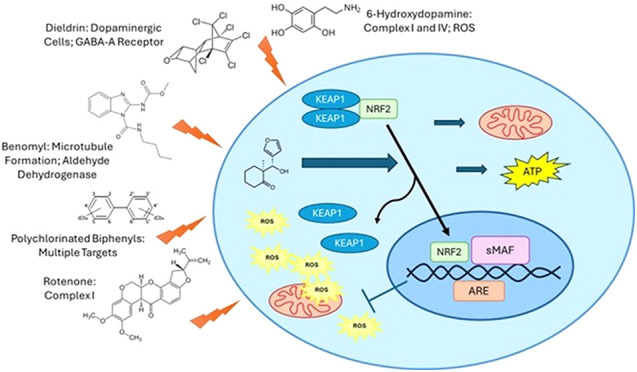

## Introduction

Environmental pollutants include a vast array of chemicals ranging from pesticides, such as benomyl and the organochlorine dieldrin, to industrial agents and byproducts, such as polychlorinated biphenyl compounds (PCB). Many of these compounds are hypothesized to be risk factors for neurodevelopmental disorders and neurodegenerative diseases, including attention deficit-hyperactivity disorder (ADHD), autism spectrum disorder (ASD), Alzheimer’s Disease (AD), and Parkinson’s Disease (PD).^1–3^ Despite the selectivity of pesticides for insects, humans remain at significant risk for neurological deficits throughout the lifespan. Chemicals, such as PCBs, found in the environment as legacy compounds, are unintentional byproducts of paint production and other industrial processes and are detected in indoor and outdoor air samples.^4,5^ The need for neuroprotective therapies to address these deficits is great, and understanding their etiology is even more critical.

In a prior report, the natural product fraxinellone was found to mitigate cell death caused by elevated levels of the endogenous toxicant glutamate (Glu) (Figure 1).^6,7^ Given this, we synthesized a library of novel fraxinellone analogs, several of which demonstrated improved protection against Glu-mediated toxicity compared to the parent compound, fraxinellone.^8^ We elucidated the mechanism of action and demonstrated that the novel, active analogs targeted nuclear factor erythroid 2-related factor (NRF2)-Kelch-like-ECH-associated protein 1 (KEAP1) signaling, yielding NRF2 translocation to the nucleus and induction of numerous protective genes, including *Nqo1*, *Sod2*, and *Gpx4*.^8^ The induction of these protective genes correlated with the rapid mitigation of Glu-mediated reactive oxygen species (ROS) generation. In particular, our previous work focused on the comparison of an inactive (analog 1) with an active (analog 2) structural analog of fraxinellone (Figure 1).^8^ Analog 2 was found to be a potent, rapid inducer of NRF2-related genes that lacks an electrophilic center (i.e., thiol-reactive) found in many currently utilized activators of the NRF2 response.^9^ A noncovalent activator of NRF2 offers advantages for therapeutic development, such as minimizing off-target interactions and improving pharmacokinetics compared to a covalent, electrophilic modifier.

**Figure 1:**
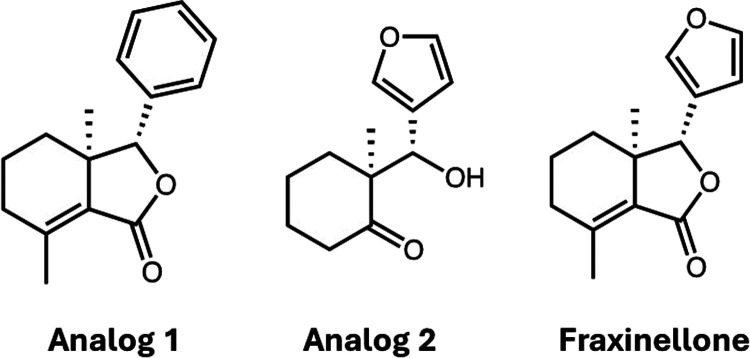
Structures of analog 1 and analog 2 along with the parent compound, Fraxinellone.

With these findings, we then sought to explore whether our fraxinellone analogs could also protect against oxidative insults beyond Glu. Our initial results showed significant activity against a high dose (1.0 μM) of rotenone, suggesting that the protection afforded by the fraxinellone analog is both generalizable and applicable to other oxidative insults.^8^ Several other reports have shown that NRF2 plays a pivotal role in several pathologies and may safeguard cells against insults to prevent disease and abnormal development.^10–12^

The question is the general applicability for protection against other known neurotoxicants with varying mechanistic targets, specifically 6-hydroxydopamine (6-OHDA), dieldrin, benomyl, 4-OH-PCB52 (PCB52 metabolite), 4-OH-PCB52 sulfate (PCB52 metabolite), and rotenone. Both rotenone and 6-OHDA are model Parkinsonian neurotoxicants, selectively affecting dopaminergic cells by targeting the mitochondrial complexes (both rotenone and 6-OHDA) and producing excessive ROS (6-OHDA).^13,14^ The organochlorine dieldrin targets GABA-A receptors and is toxic to dopaminergic cells via various mechanisms.^15–17^ Benomyl is a fungicide known to inhibit microtubule formation in pests and to inactivate aldehyde dehydrogenase.^18,19^ The PCB52 metabolites PCB52-OH and HO-PCB52 sulfate were recently shown to target mitochondria, causing a bioenergetic deficit and ROS formation.^20,21^

For the current study, we selected analog 2 as it represents an effective activator of NRF2 signaling with much higher potency compared to the parent compound fraxinellone.^8^ In addition, analog 1 was included in all studies as a negative control, given its structural similarity to fraxinellone, but it cannot interact with NRF2-KEAP1 under the conditions used.

In vitro methods were used to assess the activity of our fraxinellone analog (i.e., analog 2) against a high dose of each neurotoxicant in two cell lines, rat PC12 and human SH-SY5Y. Total cellular ROS production and its mitigation by analog 2 were measured. In addition, we visualized rotenone-mediated mitochondrial ROS production and the impact of analog 2. Our findings demonstrate that analog 2 effectively mitigated ROS production and cell death upon exposure to a high dose of all five neurotoxicants, each of which exhibits a unique mechanism of toxicity (Table 1). These results further suggest that the activity of analog 2 is generalizable, and its use could potentially be employed proactively against a wide range of environmental toxicants.

**Table 1:** Summary of Neurotoxicants and Concentrations Used for Previously Described Experiments^a^

neurotoxicant	mechanistic target	concentration used
6-OHDA	inhibits complex I and IV; ROS	high dose: 100.0 μM
dieldrin	dopaminergic cells; GABA-A receptor (insect and human relevant)	high dose: 100.0 μM
benomyl	microtubule formation (fungal cells); aldehyde dehydrogenase	high dose: 100.0 μM
4-OH-PCB52	unknown; mitochondria (in vitro)	high dose: 2.0 μM
4-OH-PCB52 sulfate	unknown; mitochondria (in vitro)	high dose: 10.0 μM
rotenone	inhibits complex I	high dose: 1.0 μM

a The mechanistic targets are human-relevant unless noted otherwise. Current data for the toxicity of PCB52 metabolites are based on in vitro experiments. The high dose selected refers to a likely nonphysiologic level that produces approximately 50% loss of cell viability over 24 h.

## Experimental Procedures

### Chemicals and Reagents

All of the LC-PCBs and their corresponding human-relevant hydroxylated and sulfated metabolites used in this study were synthesized and provided by the Synthesis Core, ISRP, as previously described.^22–25^ PCB derivatives were authenticated following published guidelines.^26^

Analogs 1 and 2 were synthesized via a new synthetic route starting from 2,6-dimethylcyclohexanone or cyclohexenone as reported in a prior publication.^8^

3-(4,5-Dimethylthiazol-2-yl)-2,5-diphenyltetrazolium bromide (MTT) (Cat. No. M5655), Complete Mini Protease Inhibitor cocktail (Cat. No. 11836153001), and the cytotoxicity detection kit (Cat. No. 11 644 793 001) were obtained from Roche (Sigma-Aldrich, St. Louis, MO). Pierce bicinchoninic acid (BCA) Protein Assay Kit (Cat No. 23227) used for protein estimations was purchased from Thermo Fisher Scientific, (Waltham, MA). Minimal Essential Medium (MEM) (Cat. No. 11095080), Dulbecco’s-Modified Eagle Medium/Nutrient Mixture F-12 (DMEM/F-12) (Cat. No. 11320033), Fetal Bovine Serum (FBS) (Cat. No. 16000044), MEM without phenol red (Cat No. 51200038), DMEM/F-12 without phenol red (Cat. No. 11039021), Hanks Balanced Salt Solution (HBSS) (Cat. No. 14025092), and Trypsin-EDTA (0.25%) (Cat. No. 25200056) were purchased from Gibco (Thermo Fisher Scientific, Waltham, MA). Penicillin–Streptomycin (P/S) containing10,000 U/ml (Cat. No. 15140122) was purchased from Life Technologies (Carlsbad, CA).

### Cell Culture

#### PC12 Cells

PC12 rat cells were obtained from American Type Culture Collection (Manassas, VA) and were grown in tissue culture flasks in RPMI 1640 supplemented with 10% horse serum, 5% fetal bovine serum (FBS), and 1% penicillin/streptomycin at 37 °C in a humidified atmosphere of 5% CO_2_. All experiments on these cells were performed between passages 9 and 12 to reduce interexperimental variability.^8^ All cells were treated with equal volumes of DMSO before harvesting for analysis.

#### SH-SY5Y Cells

SH-SY5Y human neuroblastoma cells were obtained from American Type Culture Collection (Manassas, VA) and were grown in Opti-MEM supplemented with 10% FBS, 1% MEM-nonessential amino acids, 1% penicillin/streptomycin, and 1 mM sodium pyruvate at 37 °C in a humidified atmosphere of 5% CO_2_. All experiments on these cells were performed between passages 8 and 10 to reduce interexperimental variability.^8^ All cells were treated with equal volumes of DMSO before harvesting for analysis.

### MTT Assay

Cell viability was assessed using the colorimetric reagent, 3-[4,5-dimethylthiazol-2-yl]-2,5-diphenyltetrazolium bromide (MTT) (Sigma-Aldrich). For MTT analysis, cultures were incubated in HBSS/glucose with 2 mg/mL MTT for 2 h at 37 °C. Following incubation, 0.4 mL DMSO was added to each well to solubilize the formazan product. Reduced MTT was measured on a microplate reader (Molecular Devices Spectra Max 190) at 570 nm with a reference of 650 nm.

To determine the dose–response of the cell lines for each toxicant and aid in the selection of an appropriate concentration for each insult, the following procedure was used. Cells were treated with a range of concentrations of each toxicant, varying at least 20-fold from lowest to highest value, for 24 h. MTT analysis was performed at 24 h to assess cell viability. The concentrations used for analog 1 (0.05 to 1.0 μM) and analog 2 (0.05–5.0 μM) were found to be not toxic to either cell line at 24 h via MTT (data not shown).

Based on the dose–response curves, a “high-dose” concentration for each insult that produced approximately 50% loss of cell viability over 24 h was selected. Cells were pretreated with analog 1 or analog 2 at a range of 0–1 μM for 30 min. The analogs were then washed from the cells before adding a high final concentration (yielding approximately 50% loss of cell viability over 24 h) of each neurotoxicant for 24 h (Table 1). MTT analysis was performed at 24 h to assess cell viability.

### Rescue Experiment with Rotenone

SH-SY5Y cells were plated in a 24-well plate at a density of 50,000 cells/well in 500 μL of media. Twenty-four hours later, all media were aspirated and replaced with media containing 1 μM rotenone (except the negative control). Six hours later, analog 2 was added at concentrations ranging from 0.1 to 5 μM. Sixteen hours post-Analog 2 addition (24 h post-rotenone treatment), an MTT assay was performed.

### Assessment of ROS via Live-Cell Imaging

ROS production was detected using the mitochondrial superoxide indicator, MitoSOX Red reagent (Thermo Fisher). Briefly, SH-SY5Y cells were stained with 5 μM MitoSOX for 30 min prior to 30 min treatment with 100 nM analog 1 or analog 2 and then exposure to 0.1 μM or 1.0 μM Rotenone for 4 h. In addition, cells were incubated with 2.5 μM Antimycin A as a positive control for ROS production. Following treatment, live-cell images were acquired to measure ROS production using EVOS FL Auto 2 (Thermo Fisher). Cells were stained 15 min prior to imaging with NucBlue Live ReadyProbes Reagent.

### Quantification of ROS by DCFDA Fluorescence

Cells were collected, resuspended in 10 mL fresh media containing 25 μM DCFDA, and incubated for 30 min in this solution. DCFDA was then washed from the cells. The cells were then plated in a 96-well black plate, nontreated (Thermo Fisher Scientific) and treated as previously described. Images were taken on a BioTek Synergy 2 using ex. 485 nm and em. 535/30 channel at 0, 2, 4, 8, 12, and 24 h.

### Data analysis and statistical tests

Experiments were performed at least three times independently, with three analytical replicates in each experiment. Data are represented as mean ± SEM or mean ± SD, as noted in each legend. Multiple tests were used to compare cell viability for toxicants at given concentrations. Statistical significance was initially set to *p* ≤ 0.05 and is noted in each figure legend. Data were analyzed by GraphPad Prism versions 8.3.0 and above for Windows (GraphPad Software, San Diego, CA).

## Results

### Analog 2 Protects PC12 and SH-SY5Y Cells against the Screened Toxicants (Table 1) and Mitigates ROS Production via Each Agent

#### 6-OHDA

Cell viability was assessed following treatment with 6-OHDA at a range of doses in PC12 (Figure 2A) and SH-SY5Y (Figure 2B) cells. We then sought to investigate the activity of analog 2 and analog 1 against 100 μM 6-OHDA in PC12 (Figure 2C) and SH-SY5Y (Figure 2D) cells. Cells were treated with analog 1 or analog 2 for 30 min, washed, and incubated with 6-OHDA for 24 h. Analog 2 significantly protected against 100 μM 6-OHDA at as low as 0.1 μM in PC12 cells and 50 nM in SH-SY5Y cells, whereas analog 1 did not. These results suggest that our fraxinellone analog (analog 2) protects against 6-OHDA-mediated cell death; however, higher concentrations were required to mitigate 6-OHDA-mediated cell death than those needed to protect against Glu-mediated cell death.^8^ We then sought to determine if analog 2 reduces 6-OHDA-mediated ROS production in PC12 cells, thereby providing cytoprotection. Figure 2E displays the relative fluorescence units (RFU) following 6-OHDA treatment with analog 1 and analog 2. In untreated cells, minimal accumulation of ROS occurred over the course of 24 h as measured using 2′,7′-dichlorodihydrofluorosceine diacetate (DCFDA) (Figure 2E). PC12 cells showed a significant increase in ROS as early as 4 h after exposure to 6-OHDA alone. Treatment with analog 1 prior to 6-OHDA did not attenuate oxidative stress in 6-OHDA-treated cells; however, incubation with analog 2 before the 6-OHDA insult significantly diminished ROS production as early as 2 h, indicating analog 2 rapidly reduces oxidative stress caused by 6-OHDA. Collectively, these results demonstrate that analog 2 rapidly mitigates 6-OHDA-induced oxidative stress in PC12 cells in a time-dependent manner (Figure 2E).

**Figure 2:**
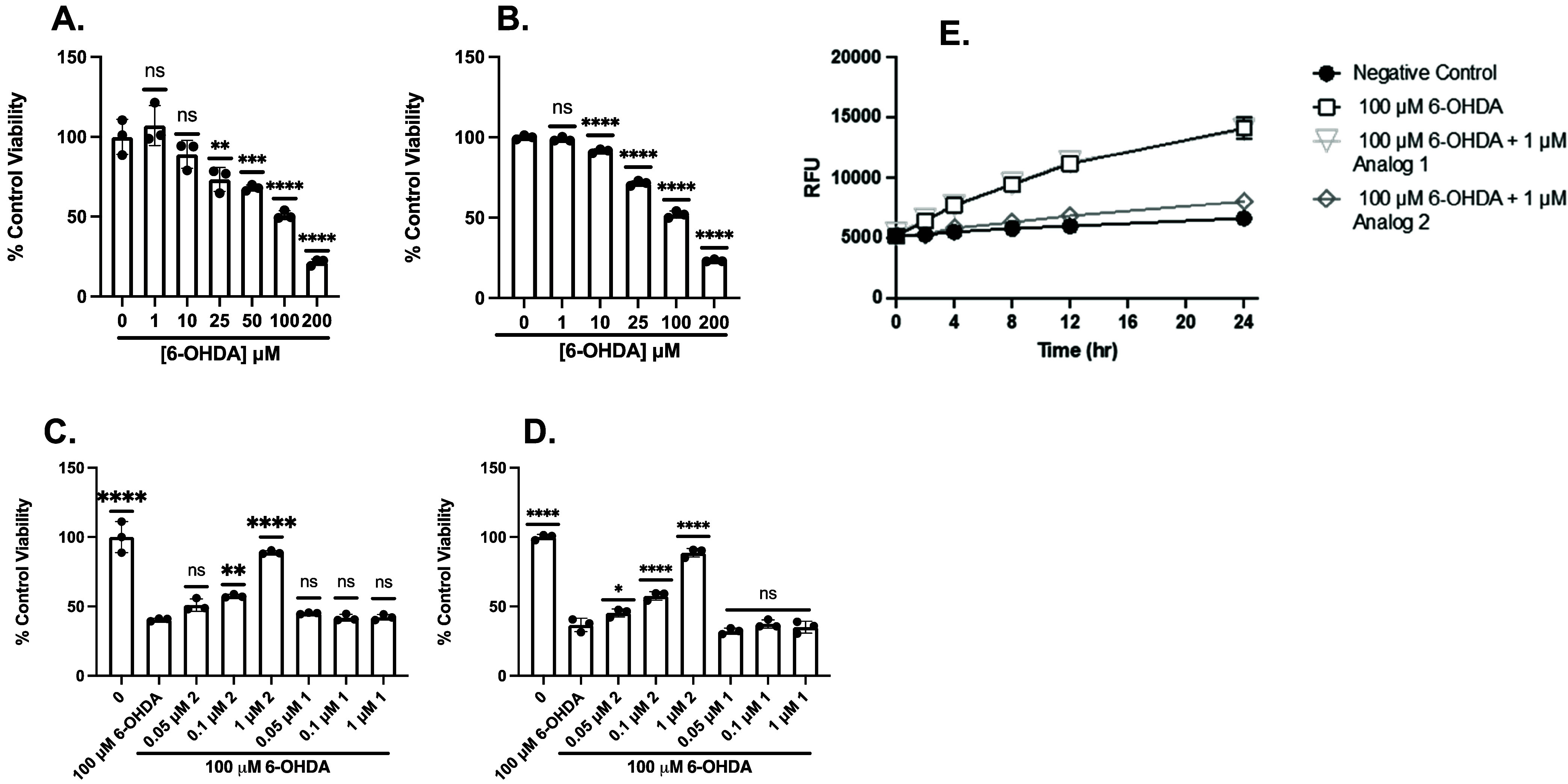
Pretreatment with analog 2 afforded protection against 6-OHDA-induced oxidative toxicity, whereas pretreatment with analog 1 did not. MTT analysis to assess cell viability of PC12 (A) or SH-SY5Y (B) treated with a range of doses of 6-OHDA for 24 h. MTT analysis to assess cell viability of PC12 (C) or SH-SY5Y (D) cells pretreated with analog 2 or analog 1 for 30 min and then 100 mM 6-OHDA for 24 h. Viability is shown as a percent of PC12 or SH-SY5Y cells left untreated. Error bars show standard deviation (SD) for *n* = 3 replicates. *p < 0.05, **p < 0.01, ***p < 0.0005, ****p < 0.0001 for ordinary one-way ANOVA comparing 6-OHDA ± analog 2 or analog 1 treated cells to untreated cells with Dunnett correction for multiple comparisons. (E) Relative Fluorescence: 6-OHDA-mediated ROS production is attenuated by 1 μM analog 2 but not 1 μM analog 1. Data shown are for mean H2DCFDA fluorescence of PC12 cells treated with 6-OHDA, pretreated with analog 1 followed by 6-OHDA, pretreated with analog 2 followed by 6-OHDA, or left untreated. Error bars show SEM for *n* = 3 replicates.

#### Dieldrin

Cell viability was assessed following treatment with dieldrin at a range of doses in PC12 (Figure 3A) and SH-SY5Y (Figure 3B) cells. The protective activity of the analogs against 100 μM dieldrin in PC12 (Figure 3C) and SH-SY5Y (Figure 3D) cells was determined. We pretreated our cells with analog 1 or analog 2 for 30 min and then washed the cells before incubating with dieldrin for 24 h. Analog 2 significantly protected against 100 μM dieldrin at as low as 0.1 μM in PC12 cells and 50 nM in SH-SY5Y cells, whereas analog 1 did not. These results suggest that our fraxinellone analogs attenuate dieldrin-mediated oxidative cell death; however, higher concentrations were required to protect against dieldrin-mediated cell death than those needed for Glu treatment.^8^ Figure 3E displays the RFU following dieldrin treatment with analog 1 and analog 2. In untreated cells, minimal ROS accumulation was observed over 24 h, as measured using DCFDA (Figure 3E); however, PC12 cells showed a significant increase in ROS as early as 2 h after dieldrin exposure alone. Treatment with analog 1 prior to dieldrin did not attenuate oxidative stress in dieldrin-treated cells; however, incubation with analog 2 before the dieldrin insult significantly decreased ROS production as early as 2 h, indicating analog 2 rapidly reduces oxidative stress caused by dieldrin. Collectively, these results demonstrate that analog 2 rapidly attenuates dieldrin-induced oxidative stress in PC12 cells in a time-dependent manner (Figure 3E). These results were confirmed using the CellTiter-Fluor assay (Figure S1).

**Figure 3:**
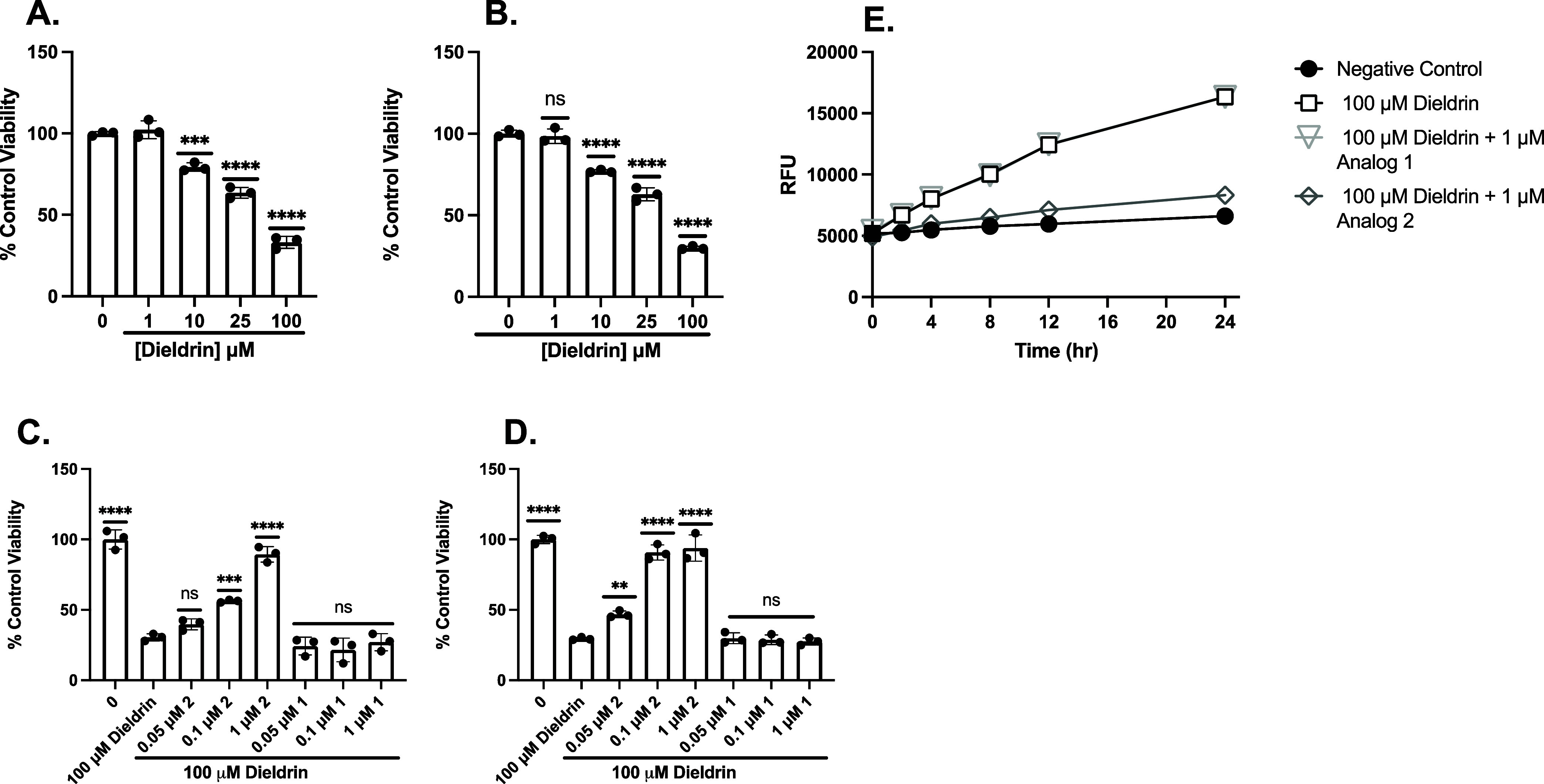
Pretreatment with analog 2 afforded protection against dieldrin-induced oxidative toxicity, whereas pretreatment with analog 1 did not. MTT analysis to assess cell viability of PC12 (A) or SH-SY5Y (B) treated with a range of doses of dieldrin for 24 h. MTT analysis to assess cell viability of PC12 (C) or SH-SY5Y (D) cells pretreated with analog 2 or analog 1 for 30 min and then 100 mM dieldrin for 24 h. Viability is shown as a percent of PC12 or SH-SY5Y cells left untreated. Error bars show SD for *n* = 3 replicates. *p < 0.05, **p < 0.01, ***p < 0.0005, ****p < 0.0001 for ordinary one-way ANOVA comparing dieldrin ± analog 2 or analog 1 treated cells to untreated cells with Dunnett correction for multiple comparisons. (E) Dieldrin-mediated ROS production is attenuated by 1 μM analog 2 but not 1 μM analog 1. Data shown are for mean H2DCFDA fluorescence of PC12 cells treated with dieldrin, pretreated with analog 1 followed by dieldrin, pretreated with analog 2 followed by dieldrin, or left untreated. Error bars show SEM for *n* = 3 replicates.

#### Benomyl

Cell viability was assessed following treatment with benomyl at a range of doses in PC12 (Figure 4A) and SH-SY5Y (Figure 4B) cells. The protection afforded by both analogs against 100 μM benomyl was determined in PC12 (Figure 4C) and SH-SY5Y (Figure 4D) cells. We treated our cells with analog 1 or analog 2 for 30 min, washed them, and incubated them with benomyl for 24 h. Analog 2 significantly protected against 100 μM benomyl at as low as 50 nM in PC12 and SH-SY5Y cells, whereas analog 1 did not. These results suggest our fraxinellone analog protects against benomyl-mediated oxidative cell death; however, higher concentrations were needed to mitigate benomyl-mediated cell death than those needed for Glu treatment.^8^ Figure 4E displays the RFU following benomyl treatment with analog 1 and analog 2. Exposure of cells to benomyl resulted in a time-dependent formation of ROS. Figure 4E displays the RFU following benomyl treatment with analog 1 and analog 2. In untreated cells, minimal ROS accumulation was observed over the course of 24 h as measured using DCFDA (Figure 4E). PC12 cells showed a significant increase in ROS as early as 2 h following benomyl exposure alone. Treatment with analog 1 prior to benomyl did not decrease oxidative stress in benomyl-treated cells; however, incubation with analog 2 before the benomyl insult significantly attenuated ROS production as early as 2 h, indicating that analog 2 rapidly reduces oxidative stress caused by benomyl. Collectively, these results demonstrate that analog 2 decreases benomyl-induced oxidative stress in PC12 cells in a time-dependent manner (Figure 4E). These results were confirmed using the CellTiter-Fluor assay (Figure S2).

**Figure 4:**
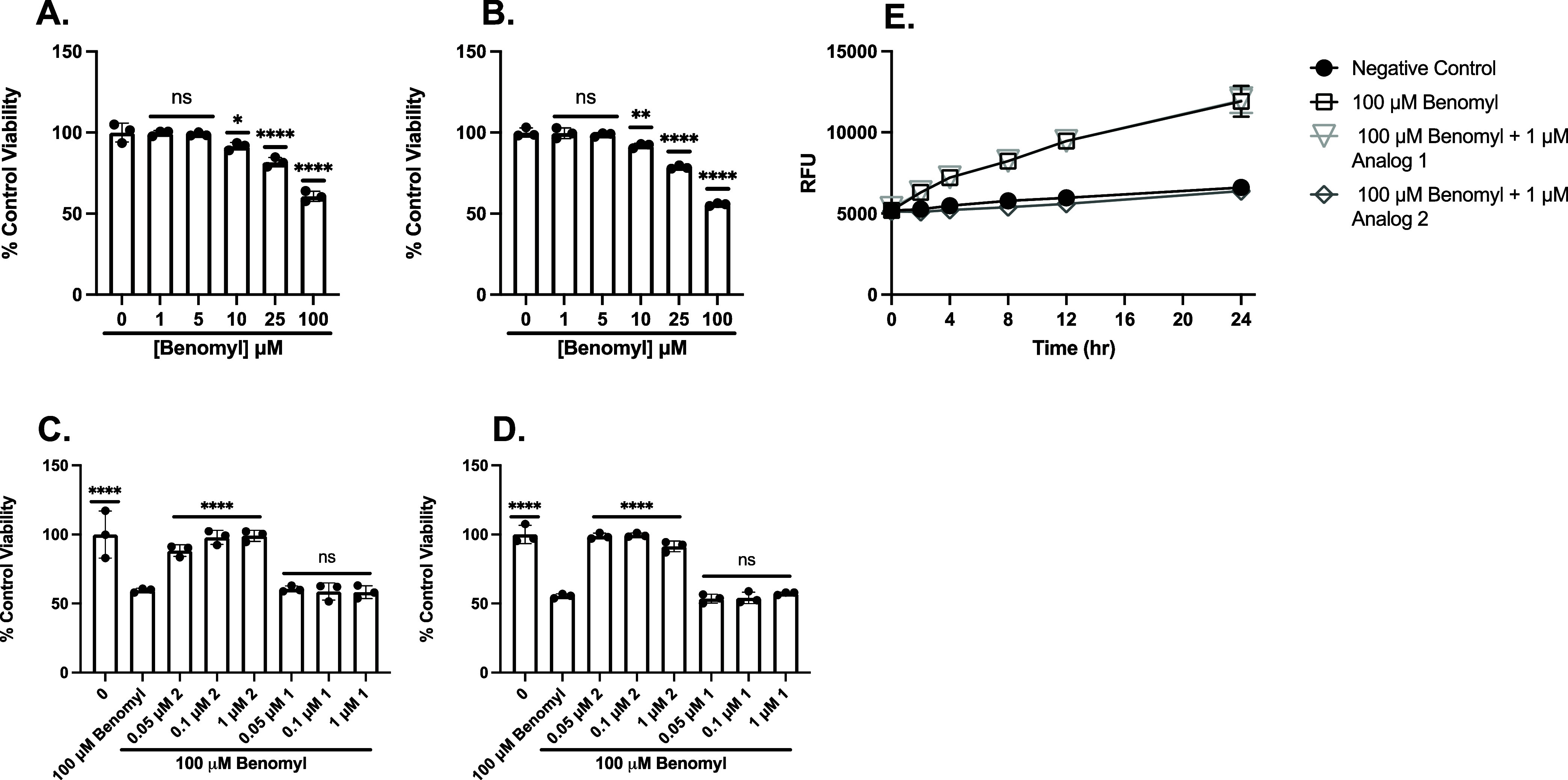
Pretreatment with analog 2 afforded protection against benomyl-induced oxidative toxicity, whereas pretreatment with analog 1 did not. MTT analysis to assess cell viability of PC12 (A) or SH-SY5Y (B) treated with a range of doses of benomyl for 24 h. MTT analysis to assess cell viability of PC12 (C) or SH-SY5Y (D) cells pretreated with analog 2 or analog 1 for 30 min and then 100 mM benomyl for 24 h. Viability is shown as a percent of PC12 or SH-SY5Y cells left untreated. Error bars show SD for *n* = 3 replicates. *p < 0.05, **p < 0.01, ***p < 0.0005, ****p < 0.0001 for ordinary one-way ANOVA comparing benomyl ± analog 2 or 1 treated cells to untreated cells with Dunnett correction for multiple comparisons. (E) Benomyl-mediated ROS production is attenuated by 1 μM analog 2 but not 1 μM analog 1. Data shown are for mean H2DCFDA fluorescence of PC12 cells treated with benomyl, pretreated with analog 1 followed by benomyl, pretreated with analog 2 followed by benomyl, or left untreated. Error bars show SEM for *n* = 3 replicates.

#### 4-OH-PCB52 and 4-OH-PCB52 Sulfate

We then investigated the activity of our fraxinellone analogs against 4-OH-PCB52 and 4-OH-PCB52 sulfate. Experiments were performed using the same methods as previously reported.^8^ Cell viability was assessed following treatment with 4-OH-PCB52 at a range of doses in PC12 (Figure 5A) and SH-SY5Y (Figure 5B) cells. We then sought to investigate the activity of analog 2 and analog 1 against 2.0 μM 4-OH-PCB52 in PC12 (Figure 5C) and SH-SY5Y (Figure 5D) cells. Cells were pretreated with analog 1 or analog 2 for 30 min and then washed before incubating with 4-OH-PCB52 for 24 h. Analog 2 significantly protected against 2.0 μM 4-OH-PCB52 at as low as 50 nM in PC12 and SH-SY5Y cells, whereas analog 1 did not. These results suggest that our fraxinellone analogs attenuate 4-OH-PCB52-mediated oxidative cell death; however, higher concentrations were required to mitigate 4-OH-PCB52-mediated cell death than those needed following Glu treatment.^8^

**Figure 5:**
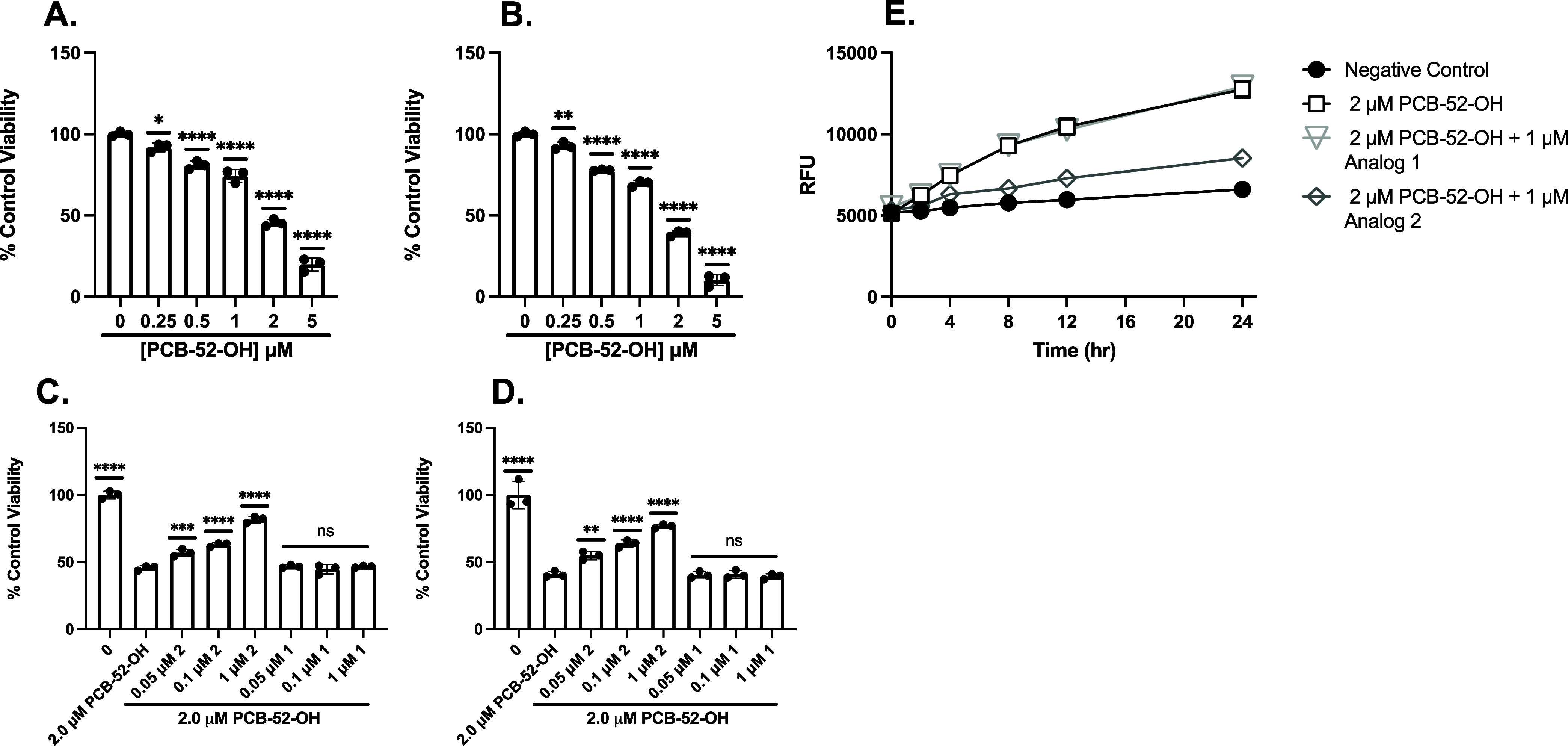
Pretreatment with analog 2 afforded protection against 4-OH-PCB52-induced oxidative toxicity, whereas pretreatment with analog 1 did not. MTT analysis to assess cell viability of PC12 (A) or SH-SY5Y (B) treated with a range of doses of 4-OH-PCB52 for 24 h. MTT analysis to assess cell viability of PC12 (C) or SH-SY5Y (D) cells pretreated with analog 2 or analog 1 for 30 min and then 2.0 mM 4-OH-PCB52 for 24 h. Viability is shown as a percent of PC12 or SH-SY5Y cells left untreated. Error bars show SD for *n* = 3 replicates. *p < 0.05, **p < 0.01, ***p < 0.0005, ****p < 0.0001 for ordinary one-way ANOVA comparing 4-OH-PCB52 ± analog 2 or 1 treated cells to untreated cells with Dunnett correction for multiple comparisons. (E) 4-OH-PCB52-mediated ROS production is attenuated by 1 μM analog 2 but not 1 μM analog 1. Data shown are for mean H2DCFDA fluorescence of PC12 cells treated with 4-OH-PCB52, pretreated with analog 1 followed by 4-OH-PCB52, pretreated with analog 2 followed by 4-OH-PCB52, or left untreated. Error bars show SEM for *n* = 3 replicates.

Figure 5E displays the RFU following 4-OH-PCB52 treatment with analog 1 and analog 2. PC12 cells showed a significant increase in ROS as early as 4 h following 4-OH-PCB52 exposure alone (Figure 5E). Treatment with analog 1 prior to 4-OH-PCB52 did not prevent oxidative stress in 4-OH-PCB52-treated cells; however, incubation with analog 2 before the 4-OH-PCB52 insult significantly attenuated ROS production as early as 2 h, indicating that analog 2 rapidly reduces oxidative stress caused by 4-OH-PCB52. Collectively, these results demonstrate that analog 2 rapidly diminishes 4-OH-PCB52-induced oxidative stress in PC12 cells in a time-dependent manner (Figure 5E).

Cell viability was assessed following treatment with 4-OH-PCB52 sulfate at a range of doses in PC12 (Figure 6A) and SH-SY5Y (Figure 6B) cells. We then investigated the activity of analog 2 and analog 1 against 10.0 μM 4-OH-PCB52 sulfate in PC12 (Figure 6C) and SH-SY5Y (Figure 6D) cells. Cells were pretreated with analog 1 or analog 2 for 30 min and then washed before incubating with 4-OH-PCB52 sulfate for 24 h.

**Figure 6:**
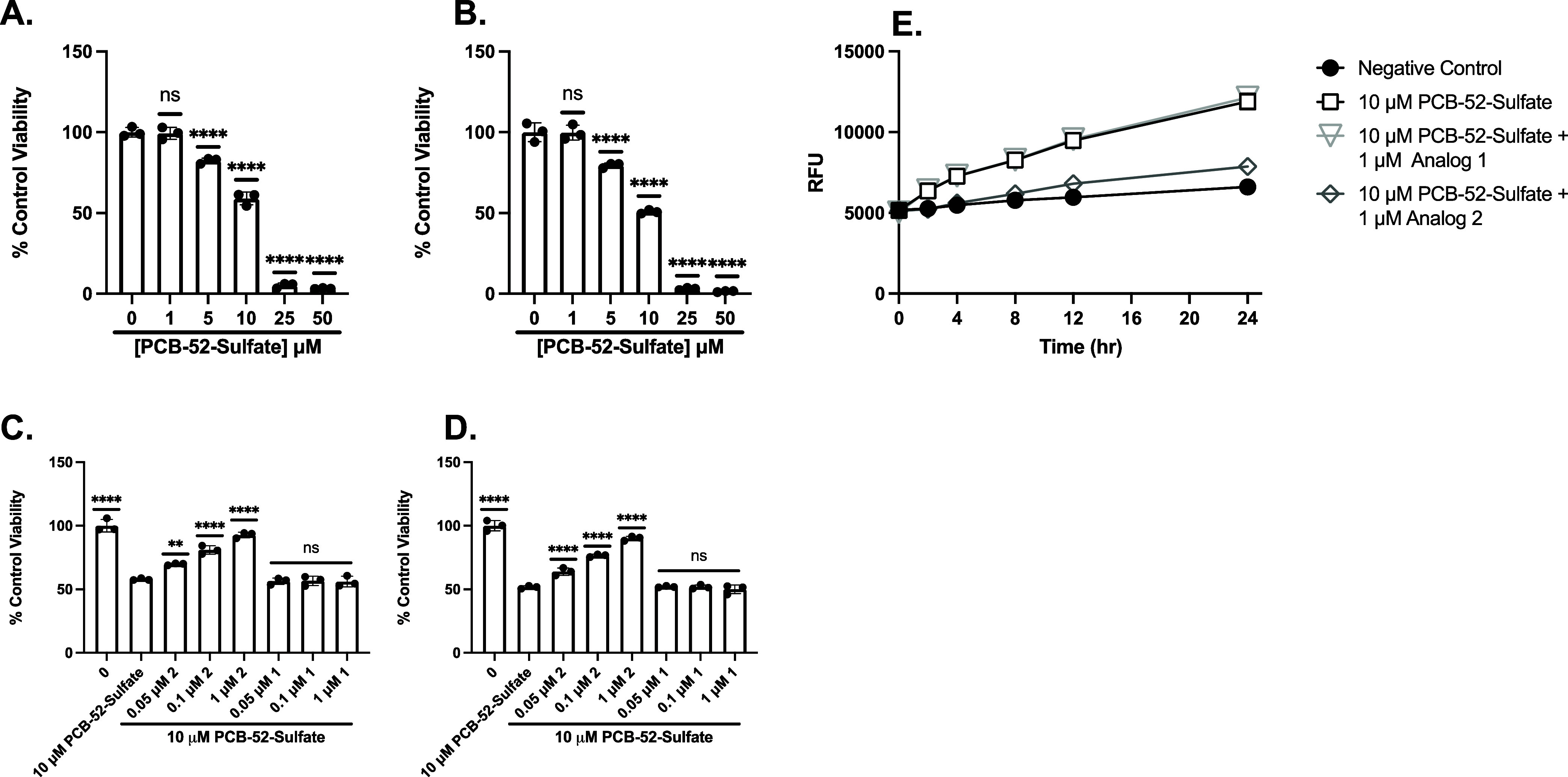
Pretreatment with analog 2 afforded protection against 4-OH-PCB52 sulfate-induced oxidative toxicity, whereas pretreatment with analog 1 did not. MTT analysis to assess cell viability of PC12 (A) or SH-SY5Y (B) treated with a range of doses of 4-OH-PCB52 sulfate for 24 h. MTT analysis to assess cell viability of PC12 (C) or SH-SY5Y (D) cells pretreated with analog 2 or analog 1 for 30 min and then 10.0 mM 4-OH-PCB52 sulfate for 24 h. Viability is shown as a percent of PC12 or SH-SY5Y cells left untreated. Error bars show SD for *n* = 3 replicates. *p < 0.05, **p < 0.01, ***p < 0.0005, ****p < 0.0001 for ordinary one-way ANOVA comparing 4-OH-PCB52 sulfate ± analog 2 or 1 treated cells to untreated cells with Dunnett correction for multiple comparisons. (E) 4-OH-PCB52 sulfate-mediated ROS production is attenuated by 1 μM analog 2 but not 1 μM analog 1. Data shown are for mean H2DCFDA fluorescence of PC12 cells treated with 4-OH-PCB52 sulfate, pretreated with analog 1 followed by 4-OH-PCB52 sulfate, pretreated with analog 2 followed by 4-OH-PCB52 sulfate, or left untreated. Error bars show SEM for *n* = 3 replicates.

Analog 2 significantly protected against 10 μM 4-OH-PCB52 sulfate as low as 50 nM in PC12 and SH-SY5Y cells, whereas analog 1 did not. These results suggest our fraxinellone analogs work against 4-OH-PCB52 sulfate-mediated oxidative cell death; however, higher concentrations were needed to mitigate 4-OH-PCB52 sulfate-mediated cell death compared to that seen following Glu treatment.^8^ Figure 6E displays the RFU following PCB52 sulfate treatment with analog 1 and Analog 2. As early as 4 h, PC12 cells showed a significant increase in ROS following 4-OH-PCB52 sulfate exposure alone. Treatment with analog 1 prior to 4-OH-PCB52 sulfate did not attenuate oxidative stress in 4-OH-PCB52 sulfate-treated cells; however, incubation with analog 2 before the 4-OH-PCB52 sulfate insult significantly reduced ROS production as early as 2 h, indicating analog 2 rapidly reduces oxidative stress caused by 4-OH-PCB52 sulfate. Collectively, these results demonstrate that analog 2 rapidly diminishes 4-OH-PCB52 sulfate-induced oxidative stress in PC12 cells in a time-dependent manner (Figure 6E).

### Analog 2 Mitigates Mitochondrial ROS Formation via Rotenone

To assess the ability of analog 2 to mitigate not only total cellular but also mitochondrial-specific oxidative stress, we used live-cell imaging with MitoSOX to visualize ROS production induced by rotenone. SH-SY5Y cells were treated with 100 nM analog 1 or analog 2 for 30 min prior to incubation with 0.1 μM or 1.0 μM rotenone. ROS was detected with MitoSOX after 4 h using 0.1 μM or 1.0 μM rotenone or 2.5 μM Antimycin A (positive control). As shown in Figure 7 and compared to vehicle control (Figure 7A) and the positive controls antimycin A (Figure 7B) and rotenone (Figure 7C,D), pretreatment with 100 nM analog 2 yielded a significant reduction in MitoSOX staining (Figure 7E,F), even less than that observed for the negative control (cells only). Pretreatment with analog 1 did not yield any decrease in MitoSOX fluorescence (Figure 7G,H). Quantitative analysis was performed to generate mean fluorescence intensity (Figure 7I). Analog 2 also mitigated not only mitochondrial but also total cellular ROS production in PC12 cells (Figure 7J). Treatment with analog 1 before rotenone did not attenuate oxidative stress in rotenone-treated cells; however, incubation with analog 2 before the rotenone insult significantly attenuated ROS production as early as 2 h, indicating analog 2 rapidly reduces oxidative stress caused by rotenone. Collectively, these results demonstrate that analog 2 rapidly attenuates rotenone-induced oxidative stress in PC12 cells in a time-dependent manner (Figure 7).

**Figure 7:**
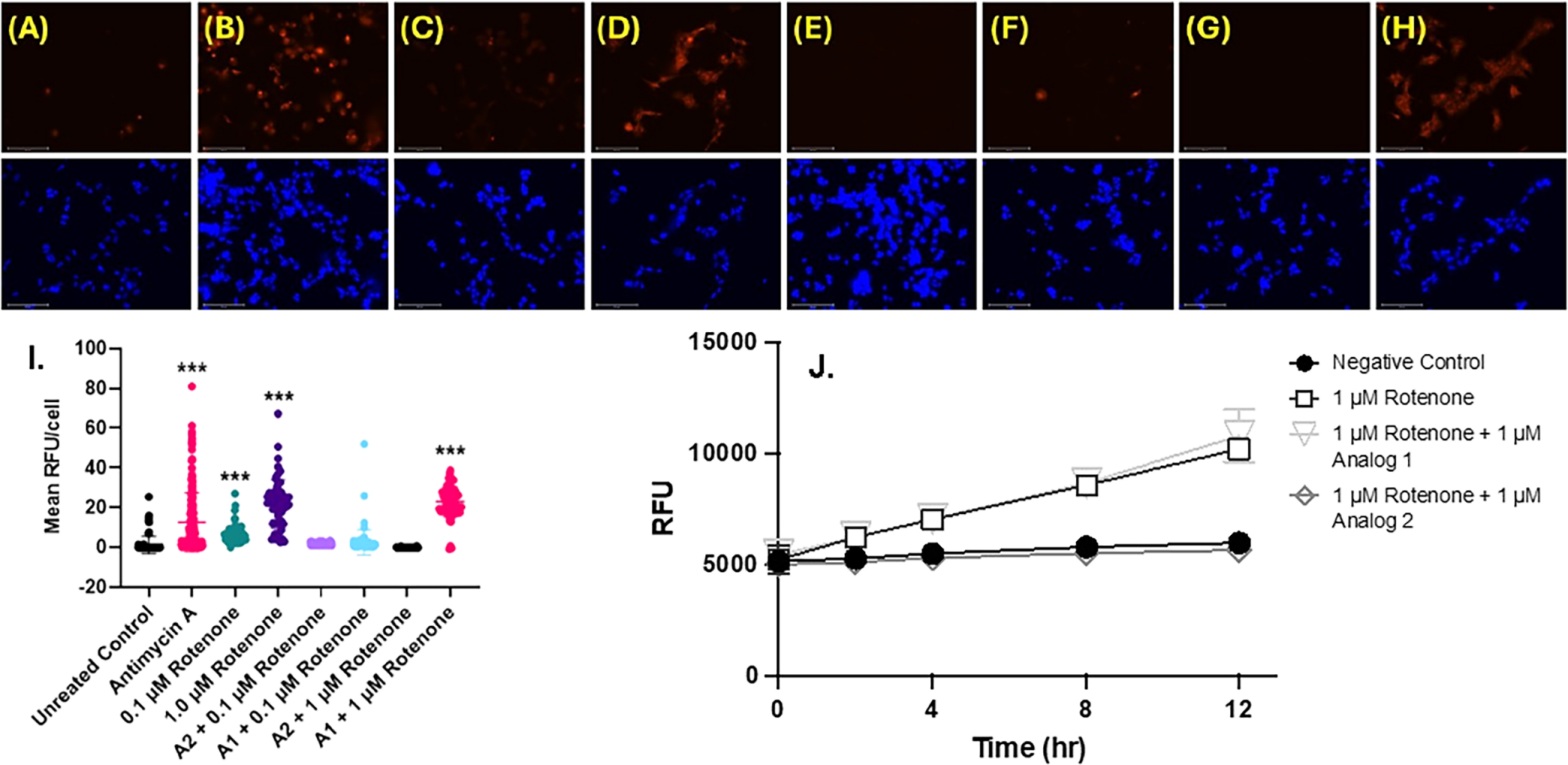
Preincubation (30 min) with analog 2 mitigates ROS production in SH-SY5Y cells treated with 0.1 or 1.0 μM rotenone for 4 h. Shown are images of cells costained with both MitoSOX (top) and DAPI (bottom) following 4 h of incubation: (A) untreated control; (B) 2.5 μM antimycin A (positive control); (C) 0.1 μM rotenone; (D) 1.0 μM rotenone; (E) 100 nM analog 2 + 0.1 μM rotenone; (F) 100 nM analog 1 + 0.1 μM rotenone; (G) 100 nM analog 2 + 1.0 μM rotenone; (H) 100 nM analog 1 + 1.0 μM rotenone; (I) Mean fluorescence intensity for each panel; ***p < 0.001 compared to untreated control. (J) Rotenone-mediated total cellular ROS production is attenuated by 1 μM analog 2 but not 1 μM analog 1. Data shown are for mean H2DCFDA fluorescence of PC12 cells treated with rotenone, pretreated with analog 1 followed by rotenone, pretreated with analog 2 followed by rotenone, or left untreated. Error bars show SEM for *n* = 3 replicates. Scale bar represents 100 μm.

### Analog 2 Restores Viability for SH-SY5Y Cells Treated with Rotenone

To determine the restorative ability of Analog 2, we incubated SH-SY5Y cells with 1 μM rotenone for 6 h, followed by the addition of 0.1–5.0 μM analog 2 for 18 h, yielding a 24 h treatment. As noted in Figure 8, there was a significant dose-dependent increase in cell viability for analog 2 compared to the positive control (i.e., rotenone) starting at 0.25 μM.

**Figure 8:**
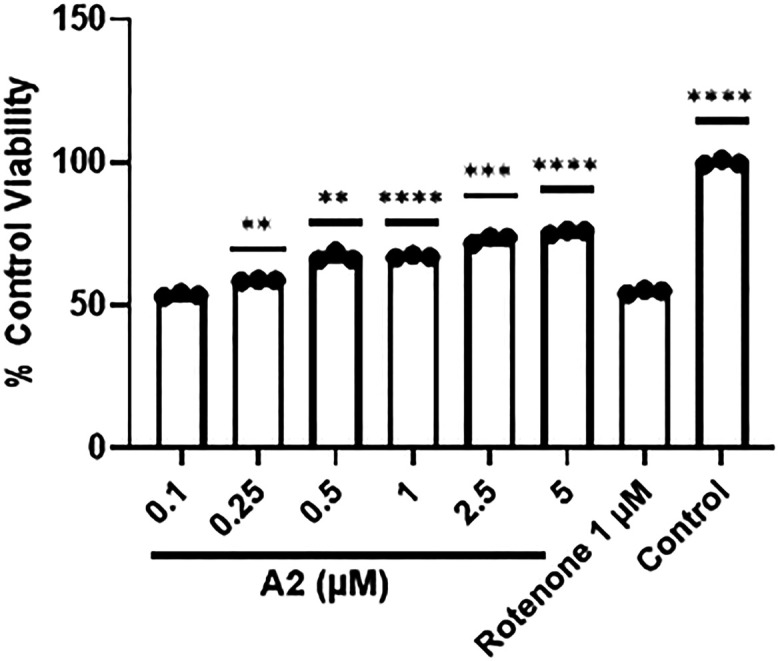
Analog 2 (A2) treatment of SH-SY5Y cells provides protection and increases viability following 1 μM rotenone treatment. Cells were incubated with rotenone for 6 h and then treated with analog 2 for 16 h, followed by analysis via MTT for viability. *p < 0.05, **p < 0.01, ***p < 0.0005, ****p < 0.0001 for one-way ANOVA comparing untreated or rotenone + analog 2 treated cells to rotenone-treated cells with Dunnett correction for multiple comparisons. Error bars show SEM for *n* = 3 replicates.

## Discussion

Our previous work established that a fraxinellone analog (i.e., analog 2) mitigated Glu toxicity in PC12 and SH-SY5Y cells by activating NRF2 signaling and inducing antioxidant and xenobiotic-metabolizing enzymes.^8^ To further assess whether the protection afforded by this fraxinellone analog is generalizable and applicable to other oxidative insults with divergent structures and varying mechanistic targets, we selected 5 known neurotoxicants and determined whether analog 2 protects 2 different cell lines against a high dose of each (Table 1). The first toxicant/toxin selected was 6-OHDA, which is routinely used in rodent models of Parkinsonism via the unilateral injection of 6-OHDA into the medial forebrain tract or striatum.^14,27^ Once inside cells via dopamine transporter (DAT) uptake, 6-OHDA inhibits complexes I and IV of the mitochondrial respiratory chain, leading to oxidative stress.^28–30^ In addition, the dopamine (DA)-derived neurotoxicant produces cellular ROS and can cause neuroinflammation upon sustained exposure. Interestingly, analog 2 but not analog 1 provided protection in both cell lines. Compared with Glu treatment, a much higher level of analog 2 was required to mitigate both toxicity and ROS formation, suggesting limitations in NRF2 signaling for certain mechanisms of toxicity.^8^

We then sought to explore the activity of our fraxinellone analogs against dieldrin, a widely used pesticide in the mid- to late 1900s.^15^ Dieldrin is one of the most persistent pesticides with a half-life of up to 25 years in the environment and 300 days in humans.^15^ Detectable amounts of dieldrin were found in the environment, even after its ban in 1974, and elevated levels were detected in the brains of PD patients relative to controls.^31,32^ While a GABA antagonist, the organochlorine appears to be more selectively toxic for DA neurons.^33^ Dieldrin alters the DA system by producing oxidative stress and increasing protein and mRNA levels of DAT and vesicular monoamine transporter (VMAT2).^16,17,34–37^ A sustained and elevated DA:VMAT2 ratio will favor higher cytosolic levels of DA, which will autoxidize to a quinone and produce ROS, yielding disrupted DA homeostasis and dopaminergic neurons more vulnerable later in life.^17^ Likely, the toxicity seen in acute assays (24 h) is not dependent on the pathological change in the DAT:VMAT2 ratio; however, dieldrin exposure produced a burst of ROS. A similar finding was seen for 6-OHDA. Compared with Glu treatment, a much higher level of analog 2 was required to mitigate dieldrin-induced toxicity and ROS formation.^8^

Benomyl, a widely used fungicide for controlling a wide range of fungal diseases affecting fruits and other crops, was selected as an additional toxicant for our studies.^38^ Mechanistically, benomyl and its metabolites prevent fungal proliferation specifically by interfering with microtubule assembly during the cell cycle.^39^ Evidence also shows that benomyl toxicity, in vitro and in vivo, is associated with aldehyde dehydrogenase (ALDH) inhibition in both the liver and brain.^40^ The inhibition of mitochondrial ALDH and dopaminergic damage was proposed as a risk factor for neurodegenerative diseases like PD.^18,19^ All levels of analog 2, from 50 nM to 1 μM, provided complete protection against a high dose of benomyl, unlike the other model toxicants. Likely, NRF2 signaling produces an efficient response to counter benomyl’s mechanism of toxicity, although the details are not clear at this point. Future studies will examine the mechanistic implications and whether NRF2 activators impact ALDH isoforms affected by benomyl.^41^

Polychlorinated biphenyls (PCBs) are persistent man-made environmental contaminants associated with neurotoxicity, leading to several learning and cognitive disabilities.^42–44^ Although commercial manufacturing of PCBs was banned in 1979, they are still inadvertently produced as byproducts from the manufacturing of consumer products (e.g., paint pigments) and found in environmental samples, including indoor and outdoor air.^4,45–48^ Several specific congeners, e.g., PCB52, are found at higher environmental levels than others.^45,47^ Recent work demonstrated that PCB metabolites, including hydroxylated and sulfated forms, exhibit greater toxicity than the parent compounds.^20,21^ PCBs and metabolites are proposed as risk factors for ASD and ADHD.^43,49–52^ For this study, 4-OH-PCB52 and 4-OH-PCB52 sulfates were selected as representatives of toxic PCB mediators, with a recent report indicating the mitochondria as a mechanistic target.^21^ Only at the highest level of analog 2 was protection afforded by analog 2. Such a finding may indicate that the diverse mechanistic targets of PCB52 metabolites are only partially addressed via the NRF2 signaling.

The variable levels of protection for analog 2 against the range of toxicants may demonstrate limitations for the NRF2 response in preventing or minimizing cellular injury and a dependence on the mechanism of action and potency for the toxicant. While 50 nM analog 2 readily mitigated benomyl-mediated toxicity (Figure 4), higher concentrations were required for significant protection against 6-OHDA and the organochlorines (Figures 2, 3, 5, and 6). For 6-OHDA and the organochlorines, full protection compared to the vehicle control was not achieved even at 1 μM analog 2. Such an observation may indicate the limit through which NRF2-induced enzymes can detoxify ROS, as, interestingly, 1 μM analog 2 was able to decrease ROS to the level of the negative control for benomyl (Figure 4E) but not 6-OHDA or the organochlorines (Figures 2E, 3E, 5E, and 6E). Differences in the capacity for xenobiotic metabolism may also factor into the potential for protection against an insult. Benomyl is rapidly metabolized via ester hydrolysis and hydroxylation, followed by glucuronide and sulfate conjugation, and in addition, the reactive isothiocyanate metabolite is readily deactivated via glutathione.^40,53^ Expression of enzymes instrumental for benomyl metabolism, as well as glutathione levels and glutathione-S-transferase, are under the control of NRF2.^54^ 6-OHDA rapidly auto-oxidizes to form ROS and reactive species, while the organochlorines, such as dieldrin, are slowly biotransformed to metabolites, which may limit the ability of NRF2 signaling to afford protection against such toxicants.^15,29,55^

Our previous work showed that analog 2 mitigated total cellular ROS production via Glu; however, we wondered if protection via analog 2 and NRF2 signaling would be afforded for mitochondrial-specific oxidative stress.^8,56,57^ Given that, we selected rotenone as a test compound, given that analog 2 protects against this insult; however, the previous work did not demonstrate the specific mechanism of protection beyond NRF2 activation. Rotenone is a neurotoxicant used in vitro and in vivo to model Parkinsonism with potent inhibition of complex I of the mitochondrial electron transport chain.^13^ Exposure to rotenone results in elevated mitochondrial ROS production and a loss of matrix NAD production. Interestingly, we found that analog 2 treatment attenuated both mitochondrial and total cellular ROS.

The experimental design included a 30 min incubation of cells with analog 2, followed by a wash step and treatment with each toxicant. Either Analog 2 exhibits rapid cellular uptake and intracellular accumulation, or the NRF2 pathway is quickly activated by the fraxinellone analog. Previous work has demonstrated that dissociation of NRF2-KEAP1 and nuclear translocation, followed by induction, occurs promptly in response to an oxidative insult.^8^ At this point, the kinetics and cellular partitioning for analog 2 are not known, and it remains to be determined whether earlier or later treatment than the current paradigm would further attenuate adverse outcomes.

A limitation of this study is the unknown pharmacokinetic profile of analog 2 and related compounds, with a need for in vivo validation. Furthermore, we do not know the potential for analog 2 to cross the blood–brain barrier, whether this occurs efficiently or in a manner that would allow in vivo neuroprotection for the central nervous system. Work is in progress to determine the in vivo efficacy and potency of analog 2 and related compounds, and of interest is uptake by the central nervous system. Analysis via Swiss ADME predicts a consensus (average) *c* log *P* of 1.87 and both blood-brain barrier penetration and gastrointestinal uptake via the BOILED-egg model.^58,59^ One characteristic that offers analog 2 an advantage over NRF2 activators currently used in the clinic or clinical trials is the apparent noncovalent interaction between the compound and NRF2-KEAP1, yielding release of the transcription factor NRF2, which could improve pharmacokinetic profiles, including uptake by the CNS.^9^

Interestingly, treatment of cells with analog 2 after the rotenone insult increased viability compared with the positive control (i.e., rotenone). Likely, this result indicates analog 2-mediated protection by preventing further rotenone toxicity in SH-SY5Y cells over 18 h; however, the viability of cells treated with [analog 2] at greater than 0.25 μM is significantly higher than that of the positive control. Such data indicate not only protection but also what appears to be restoration. At this point, the mechanism is unknown but could be due to NRF2-mediated increase in mitochondrial activity in surviving cells.^56,57^ LAMP2A and Bcl-2 are under the control of NRF2, and therefore, an increase in autophagy and/or inhibition of apoptosis may play a role, respectively.^54^ Previous work has shown NRF2 signaling yields induction of Bcl-2 with a decrease in Bax and Caspase 3/7 activity.^60^ Therefore, analog 2 treatment may be conferring a pro-survival phenotype to cells via multiple pathways (i.e., mitochondria energetics, autophagy, and antiapoptosis).

Protection against inflammation, a common endpoint from toxins/toxicants used in this study, such as 6-OHDA, was not tested in the experimental paradigm.^61^ Previous work has shown crosstalk between NRF2 and NFκB pathways.^54,62,63^ Future work will address the potential for our analogs to quell inflammatory events via NRF2-mediated interference with NFkB signaling, as well as identification of protective pathways impacted by our analogs compared to classic NRF2 activators, such as sulforaphane.

In theory, an agent such as analog 2 could be provided to populations at risk for exposure to environmental contaminants, such as those used in the current study. Many of the antioxidant enzymes induced by NRF2 have a significant biological half-life (>24h), thereby conferring protection for some manner of time before further induction is needed.^54^ Given the protective response to analog 2 appears rapid (e.g., mitigation of ROS within hours, Figure 2E), it is conceivable that analog 2 or an analog would be beneficial as an antidote.

A valid concern is overactivation of NRF2 signaling, which is a known feature of certain cancers.^64,65^ In addition, excessive induction of genes under the control of NRF2 will change drug pharmacokinetics, including the increased deactivation of anticancer therapy, and therefore, the use of NRF2 activation prophylactically or for significant periods of time has notable considerations.

Collectively, these data demonstrate the potential of analog 2, a novel fraxinellone analog, to mitigate ROS formation and toxicity induced by a panel of toxicants with diverse structures and molecular targets. A short treatment with analog 2 in 2 different cell lines afforded variable protection against the agents used in this study, likely via scavenging total cellular and mitochondrial ROS; however, other mechanisms may be at play. Future work will elucidate the kinetics and temporal window of protection as well as explore further prospects for neuroprotection offered by this novel fraxinellone analog, including translational animal studies to determine efficacy in vivo.

## Supplementary Material



## Notes

Analog 2-mediated protection against dieldrin (Figure S1) and benomyl (Figure S2) using an orthogonal cell viability assay. The methods to obtain such data are included. (PDF)

## Abbreviations

6-OHDA: 6-hydroxydopamine
A1: analog 1
A2: analog 2
AD: Alzheimer’s disease
ADHD: attention deficit-hyperactivity disorder
ASD: autism spectrum disorder
ALDH: aldehyde dehydrogenase
ARE: antioxidant response element
ATCC: American Type Culture Collection
BCA: bicinchoninic acid
DA: dopamine
DAT: dopamine transporter
DCFDA: 2/,7′-dichlorodihydrofluoresceine diacetate
DMEM/F-12: Dulbecco’s-modified Eagle medium/nutrient mixture F-12
FBS: fetal bovine serum
Glu: glutamate
HBSS: Hanks balanced salt solution
IC_50_: inhibitory concentration 50
KEAP1: Kelch-like-ECH-associated protein 1
MEM: minimal essential medium
MTT: 3-(4,5-dimethylthiazol-2-yl)-2,5-diphenyltetrazolium bromide
NRF2: nuclear factor erythroid 2-related factor
P/S: penicillin–streptomycin
PCBs: polychlorinated biphenyls
PD: Parkinson’s disease
RFU: relative fluorescence unit
ROS: reactive oxygen species
SD: standard deviation
SEM: standard error of the mean
S-MEM: minimal essential medium with Earle’s salts for suspension cultures VMAT2 vesicular monoamine transporter 2
VMAT2: vesicular monoamine transporter 2
