# Supplementary material for: Neuroprotection Against a Panel of Toxicants via a Novel Analog of the Natural Product Fraxinellone
Source: Chem Res Toxicol. 2026 Apr 22;39(5):908–19. doi: 10.1021/acs.chemrestox.5c00522 (PMC13188060; doi:10.1021/acs.chemrestox.5c00522)
Supplement: Supplementary file 1 [file tx5c00522_si_001.pdf]

Supporting Information

Neuroprotection Against a Panel of Toxicants via a Novel Analog of the Natural Product Fraxinellone

Anna E. Bartman[1], Michael A. Garcia-Mares[1], Sarah E. Preston[1], Mersad Raeisi[2], Clarence D. Peiris[2], David B.C. Martin[2], Hans-Joachim Lehmler[3], Jonathan A. Doorn[1,*]

Email: jonathan-doorn@uiowa.edu

[1]Department of Pharmaceutical Sciences and Experimental Therapeutics, College of Pharmacy, University of Iowa, Iowa City, Iowa 52242, United States.

[2]Department of Chemistry, College of Liberal Arts and Sciences, University of Iowa, Iowa City, Iowa 52242, United States.

[3]Department of Occupational and Environmental Health, College of Public Health, University of Iowa, Iowa City, Iowa 52242, United States.

Table of Contents

Supplementary Figures **S3**

Supplementary Methods **S4**

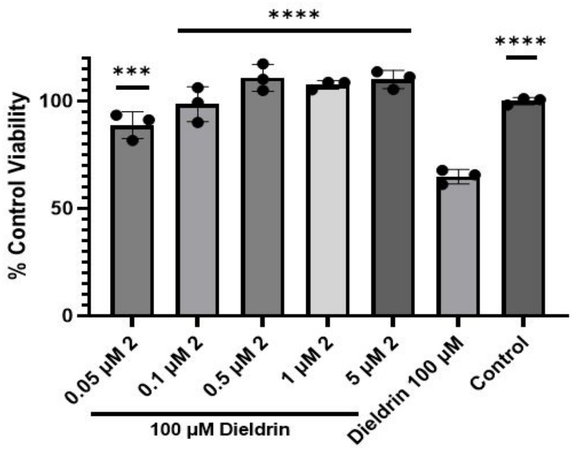

Supplementary Figure S1. Pre-treatment with Analog 2 afforded protection against dieldrin toxicity. The CellTiter-Fluor assay was used to assess cell viability of SH-SY5Y treated with Analog 2 for 30 min and then 100 mM dieldrin for 24 hr. Viability is shown as a percent of SH-SY5Y cells left untreated. Error bars show standard deviation (SD) for *n*=3 replicates. **p < 0.05, **p< 0.01, ***p< 0.0005, ****p<0.0001* for ordinary one-way ANOVA comparing dieldrin +/- Analog 2 or 1 treated cells to untreated cells with Dunnett correction for multiple comparisons.

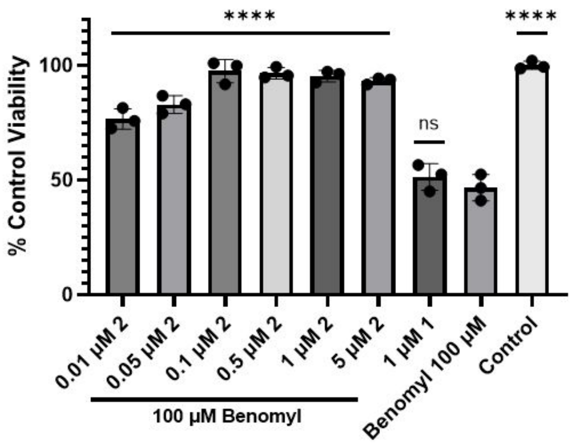

Supplementary Figure S2. Pre-treatment with Analog 2 afforded protection against benomyl toxicity whereas pre-treatment with Analog 1 did not. The CellTiter-Fluor assay was used to assess cell viability of SH-SY5Y treated with Analog 2 or Analog 1 for 30 min and then 100 mM benomyl for 24 hr. Viability is shown as a percent of SH-SY5Y cells left untreated. Error bars show standard deviation (SD) for *n*=3 replicates. **p < 0.05, **p< 0.01, ***p< 0.0005, ****p<0.0001* for ordinary one-way ANOVA comparing benomyl +/- Analog 2 or 1 treated cells to untreated cells with Dunnett correction for multiple comparisons.

Supplementary Methods

SH-SY5Y cells were plated in a 96-well plate at 25,000 cells per well in 100 µL media. 24 hours later, cells were treated for 30 minutes with A1, A2, or media. Treatment media was then aspirated and replaced with 100 µM benomyl or 100 µM dieldrin in phenol red free media. The CellTiter-Fluor (Promega, Madison, WI) cell assay was used to measure viability 24 hours later. The fluorogenic substrate glycylphenylalanyl-aminofluorocoumarin (GF-AFC) was directly added, and after a 2-hour incubation at 37°C, fluorescence (380 ex, 505 em) was read.
